# Additional perspectives on chronic kidney disease of unknown aetiology (CKDu) in Sri Lanka – lessons learned from the WHO CKDu population prevalence study

**DOI:** 10.1186/1471-2369-15-125

**Published:** 2014-07-28

**Authors:** Jennifer Hoponick Redmon, Myles F Elledge, Donna S Womack, Rajitha Wickremashinghe, Kamani P Wanigasuriya, Roshini J Peiris-John, Joseph Lunyera, Kristin Smith, James H Raymer, Keith E Levine

**Affiliations:** 1 RTI International, Center for Health and Environmetnal Modeling, 3040 East Cornwallis Rd, Research Triangle Park, NC 27709-2194, USA; 2 RTI International, Discovery Science Technology, 3040 East Cornwallis Rd, Research Triangle Park, NC 27709-2194, USA; 3 Duke University, Global Health Institute, Durham, NC, USA; 4 Department of Public Health, University of Kelaniya, Kelaniya, Sri Lanka; 5 Department of Medicine, University of Sri Jayewardenepura, Nugegoda, Sri Lanka; 6 University of Auckland Epidemiology & Biostatistics Auckland, New Zealand

**Keywords:** Chronic kidney disease, Chronic kidney disease of unknown aetiology, Environmental nephrotoxins, Sri Lanka, Heavy metals, Agrochemicals, Cadmium

## Abstract

The recent emergence of an apparently new form of chronic kidney disease of unknown aetiology (CKDu) has become a serious public health crisis in Sri Lanka. CKDu is slowly progressive, irreversible, and asymptomatic until late stages, and is not attributable to hypertension, diabetes, or other known aetiologies. In response to the scope and severity of the emerging CKDu health crisis, the Sri Lanka Ministry of Health and the World Health Organization initiated a collaborative research project from 2009 through 2012 to investigate CKDu prevalence and aetiology. The objective of this paper is to discuss the recently published findings of this investigation and present additional considerations and recommendations that may enhance subsequent investigations designed to identify and understand CKDu risk factors in Sri Lanka or other countries.

## Introduction

Sri Lanka recently transitioned from a low- to middle-income nation, and its disease pattern has shifted from infectious and maternal/childhood diseases towards non-communicable diseases (NCDs). The prevalence and associated mortality of chronic kidney disease (CKD) has been on the rise in Sri Lanka over the last two decades with this trend being exacerbated in the last decade by the emergence of an apparently new form of CKD of unknown aetiology (CKDu). Notably, CKDu is not attributed to hypertension, diabetes, or other aetiologies typically associated with traditional CKD. This emerging disease is slowly progressive, irreversible, and asymptomatic until late stages with histopathological features identified by renal biopsies indicating tubulointerstitial fibrosis and tubular atrophy [1].

CKDu is most pronounced in Sri Lanka’s North Central Province (NCP) and primarily affects people of low socioeconomic status, particularly those involved in farming or living in agricultural areas [2]. The absence of typical CKD risk factors, the geographical distribution of the disease, its prevalence among farming communities and histopathology findings collectively suggests the involvement of exposure to one or more environmental nephrotoxicants in the genesis and progression of CKDu [2].

In an effort to determine the extent and causes of CKDu in Sri Lanka, a number of aetiological studies have been conducted over the past several years. These include, laboratory studies that assessed the level of aceytlcholinesterase inhibition from possible exposure to organophosphate pesticides and screening of regional food supply for the presence of ochratoxin, a common mycotoxin [3,4]. Several studies have also focused on the assumption that heavy metals in the environment or dietary items (e.g., rice and vegetables), most notably cadmium and arsenic, may originate from the application of agrochemicals [5,6]. Some investigators have also speculated that the unique climate and hydrogeochemistry (hard water with elevated fluoride levels) in the NCP may contribute to the pathogenesis of CKDu [7].

Other investigators ponder whether dehydration is a major risk factor for CKDu given the extremely warm temperatures in the region, extensive agricultural work-related heat exposure, and local hydration habits. It is surmised that recurrent episodes of low-grade dehydration-induced kidney injury could potentially lead to kidney fibrosis [8,9]. The progression of CKDu has also been linked to Ayurvedic medicine usage or genetic susceptibility in some studies [10,11].

In response to the scope and severity of the emerging CKDu health crisis, the Ministry of Health (MoH) in Sri Lanka and the World Health Organization (WHO) initiated a collaborative research project in 2009 to investigate the prevalence and aetiology of the disease, with the ultimate goal of developing appropriate preventative strategies [12]. A critical component of this investigation was a population prevalence study to assess the scope of CKDu in selected locations defined as endemic and non-endemic regions in Sri Lanka. This case–control study evaluated the concentrations of selected heavy metals in environmental and biological samples, as well as selected pesticides in biological samples. The findings were summarized in a recent publication [13].

Jayatilake and colleagues on the Sri Lankan CKDu National Research Project Team make an important contribution by drawing greater attention to this emerging health issue and initiating the effort to obtain greater understanding of the disease prevalence. The authors used an array of analytical techniques to analyze biological and environmental samples from endemic and non-endemic CKDu areas and conclude that multiple agents may play a role in the genesis and progression of CKDu, including chronic exposure to low levels of cadmium through the food chain and also to pesticides, and that these exposures may be exacerbated by genetic factors or selenium deficiency.

Moving forward, there are opportunities to build on the research by Jayatilake et al. and other investigators who have been working to identify potential risk factors associated with CKDu in Sri Lanka. The objective of our paper is two-fold: 1) to provide a critical review of the Jayatilake et al. findings reflecting what is currently known from global literature and 2) to identify and discuss recommendations for enhancing subsequent investigations on the potential risk factors associated with CKDu in Sri Lanka or other countries. Our review presented below focuses on the following components of the Jayatilake et al. study*:* the CKDu case definition, endemic and non-endemic geographic area selection, population sampling design, multimedia sample selection, chemical constituent selection, field documentation, and laboratory analytical methodologies employed by Jayatilake and colleagues.

### CKDu case definition

Jayatilake et al. define a CKDu case as an individual identified with an albumin-to-creatinine ratio (ACR) ≥ 30 mg/g urine during initial visit and at a follow-up visit, a normal glycosylated hemoglobin (HbA_1c_ <6.5%), not on treatment for diabetes, no elevated blood pressure, and no past history of kidney disease or snake bite. The authors provided a clear case definition of CKDu and its associated disease grades. There is, however, a likelihood that false negative measurements excluded potential CKDu patients or erroneously included patients with traditional CKD.

An elevated ACR (or proteinuria) is the most widely used marker for identifying kidney damage as it is highly sensitive in the earlier stages of traditional CKD. ACR is also very cost-effective for population-based studies and thus has gained popularity as the primary indicator in community-based CKD surveys and screening activities [14]. Nevertheless, there is growing evidence that this parameter is not ideal for CKDu diagnosis, as CKDu cases typically present with minimal, if any, proteinuria in the early stages of the disease. Because Jayatilake et al. used ACR as the initial screening parameter in their case definition it is possible that a number of CKDu patients without proteinuria were inadvertently excluded.

A primary parameter that is more sensitive in screening CKDu cases is recommended to replace ACR in the CKDu case definition and the staging criteria following CKDu diagnosis. The staging criteria most frequently used for CKD is the Kidney Disease Outcomes Quality Initiative (KDOQI) CKD Guidelines from the National Kidney Foundation (NKF) [15]. Based on these guidelines, the primary parameter for case definition (diagnosis) and staging is the estimated glomerular filtration rate (eGFR), which estimates the amount of blood passing through glomeruli in the kidneys per minute. The secondary parameter is evidence of kidney damage, which serves to distinguish CKD from other causes of decreased eGFR such as diet or rhabdomyolysis (excessive breakdown of muscle).

Markers of kidney damage that are sensitive to tubular injury, such as urinary neutrophil gelatinase-associated lipocalin (NGAL)-to-creatinine ratio, may be more suitable for early diagnosis of CKDu than ACR [9]. Leakage of albumin is caused by the involvement of the glomeruli in the initial disease mechanism in traditional CKD. Evidence exists to suggest that the initial disease mechanism in CKDu primarily involves the renal tubulo-interstitium, rather than the glomeruli [1]. A number of studies have also shown that NGAL is an accurate biomarker of kidney injury [16]. Measuring urinary NGAL-to-creatinine ratio could also act as a marker of tubular injury. The efficacy of NGAL as a marker of kidney damage is discussed in several recent nephrology studies with one study demonstrating the efficacy of NGAL in the diagnosis of early CKD stages for diabetic patients that tested negative with ACR [17]. As toxin-associated kidney injury primarily affects the tubules (TIN), not the glomeruli, NGAL may be a better marker for early evidence of kidney damage in CKDu [9]. As such, it is recommended as a more sensitive primary parameter for the CKDu case definition and staging compared to ACR, which should be considered during study design for future investigations.

Another criterion to consider for CKDu diagnosis is a person’s past history of glomerulonephritis (GN). A concern is that many patients suffer episodes of GN but may not be hospitalized (or are hospitalized but misdiagnosed). Given the current and limited understanding of the CKDu causation mechanism, it is recommended to exclude patients based on their previous history of infections known to be associated with GN (particularly in areas known to have a high burden of these infectious processes), rather than to exclude based on history of GN per se. We suggest that a study should be done to test for the sensitivity and specificity of the case definition with respect to these infectious processes using the gold-standard biopsy-based diagnosis of CKDu [18].

The rationale for excluding diabetes mellitus (DM) is considered appropriate [19]. In future research efforts, hypertension (HTN) cut-off points for blood pressure (BP) are recommended to be set slightly lower (e.g. 140/90 only, rather than 160/100 if on HTN medicine), as most CKDu patients have reported BPs within the normal range [1]. Adjusting this criterion will help to further ensure that patients with CKD secondary to HTN are excluded.

### Endemic and non-endemic geographic area selection

Determining which geographic areas to include in a study is critical to its overall relevance. The authors define that three endemic districts (Anuradhapura, Polonnaruwa, and Badulla) were chosen to evaluate CKDu patient cases, controls and environmental samples. In addition, one non-endemic area was chosen (Hambantota) to evaluate control cases and environmental samples. The authors state that individuals were initially randomly recruited from six divisional areas within the three endemic districts, with 22 villages and 100 households from each of those villages randomly selected for the study. Jayatilake et al. also note that Hambantota was chosen as a non-endemic control area because CKDu “has not been reported”. In a recent publication, Chandrajith et al. [10] report that the prevalence of CKDu was surveyed in Hambantota in 2008 using proteinuria as an indicator. Based on these survey results, and similarities in the dry climate and socioeconomic background, Hambantota was deemed to be an appropriate area for a geographic control by Chandrajith et al. As noted in the CKDu case definition discussion above, proteinuria is likely not an ideal indicator for CKDu diagnosis; thus the CKDu prevalence results from this earlier survey may be under-reported. The type, quality, and access to medical care across different regions may also result in variable diagnosis and reporting procedures, potentially leading to under-diagnosis of more severe CKDu stages, non-diagnosis for lower stage CKDu cases, or misdiagnosis with traditional CKD [1]. As an aid to future research, we recommend that verification of Hambantota as a non-endemic CKDu control area is established by conducting a follow-up screening survey [20,21].

Further defining the endemic and non-endemic areas with specific exclusion criteria based on study-specific quantitative laboratory and survey data is also preferable in future studies [10,22]. Previous studies suggest that the potential for selection bias during case–control studies is present if controls do not sufficiently match the stipulated criteria and confounding factors are not taken into account [23,24]. Controls from within the endemic area are theoretically presumed to be subject to the same type of risk factors as cases within the endemic area, but with lesser severity, compared to non-endemic controls that may not be subject to the same risk factors in any amount. It is important for the authors to document these potential spatial and value differences amongst endemic and non-endemic controls during their statistical analysis. For example, based on the article results, median cadmium levels in soil from endemic and non-endemic areas do not appear to differ significantly, but large differences may exist within an area or village.

Erik von Elm et al. [25] provides an observational reporting checklist that includes eligibility criteria and control selection standards during methods documentation. Several other studies also discuss how to strengthen reporting in observational epidemiological studies using an organized writing approach or tools to assess quality and potential bias during study design or completion [26,27].

Global positioning devices and geographic information systems (GIS) tools were used during the Sri Lanka MoH and WHO study, though Jayatilake et al. did not publish details about the geographic characteristics associated with each area (e.g. geology, climate, socioeconomic status, occupations). Publication of the GIS data will help enable visualization of quantitative results (e.g. locations with elevated metals concentrations), identify the proximity of cases and controls to potential areas of interest (e.g. farms with recent agrochemical applications), and allow for a more comprehensive evaluation of potential confounding factors during quantitative statistical analysis or the qualitative interpretation of results [28-31]. For example, based on the supplementary data, cadmium levels in soil appear to be similar in both endemic and non-endemic areas. However, without spatial definition, it is difficult to draw conclusions from this similarity. The use of GIS for results visualization and interpretation will bolster the overall findings and more clearly pinpoint the risk factors associated with CKDu.

### Population sampling design

Jayatilake et al. used stratified random sampling for cases and controls. The authors note that they randomly selected district type, followed by village type, then household type within the endemic area (and non-endemic area for additional controls), to capture data on the overall prevalence of CKDu at the time of the study. Interviewers completed a questionnaire for each case and control that collected information on “age, sex, marital status, education, occupation, smoking, alcohol consumption, current residence, duration of residence in the study area, source of drinking water, storage containers for drinking water, exposure to agrochemicals, history of snake bite, glomerulonephritis, pyelonephritis, renal calculi, use of medications including herbal medicines, and past medical history”. To improve interpretation and understanding of the collected data, it is recommended that future studies include additional information regarding sample design and the questionnaire, such as what constitutes protection from agrochemicals (e.g. certain types of personal protective equipment).

The stratified random sampling performed in the study should theoretically result in an unbiased mean estimate for the overall population; however, individuals most at risk from CKDu are those with the greatest personal exposure to the disease’s potential risk factors (e.g. environmental contamination, genetic predisposition, and/or subpopulation sensitivity). Therefore, when attempting to identify what risk factors are indeed associated with CKDu, the individuals of greatest concern are expected to fall within the population maximum (also known as a hot spot) rather than the mean. It is presumed that these individuals will characteristically exhibit the greatest environmental exposure, genetic susceptibility and/or sensitivity to the risk factors associated with CKDu. This hot spot sampling approach could have been considered as an alternative sampling methodology. It is often used to characterize and delineate areas with the greatest severity and extent of environmental contamination and associated exposure pathways at Superfund sites in the United States [32].

Hot spot sampling in areas with greater reporting of CKDu prevalence (“Ground Zero”) could provide a stronger preliminary link to CKDu risk factors and the increased incidence of CKDu in an area over time. From a statistical perspective, this approach could be conducted via judgmental sampling, where prior information and surveys are deployed to hone in on areas hypothesized to be of greater concern; or via adaptive cluster sampling, where samples are first picked randomly, but after initial sample results, the sampling design will adapt to areas of greater concern based on the initial screening. Therefore, an alternative sampling approach is apt to identify the potential risk factors associated with CKDu, rather than its prevalence. After a better understanding of the risk factors associated with CKDu, a comprehensive study could be designed and implemented to obtain a representative understanding of the prevalence of CKDu.

### Multimedia sample selection

The authors are commended for undertaking a study that incorporates a wide variety of environmental and biological media samples in an effort to link the biological underpinnings of CKDu with potential environmental risk factors. This study is the most comprehensive CKDu study in Sri Lanka to-date. The biological sample types included urine, blood serum, hair and nails. The environmental sample types included water, food (e.g. rice, vegetables, and fish), fodder (e.g. pasture, weeds), tobacco, pesticides, and fertilizer. While samples were collected for each of these sample media, the number of samples varies by media type. As an example, for biological samples there were 733 CKDu cases, but between 57 and 495 individuals are chosen for urine, serum, hair, and nail samples with most individuals only tested for three heavy metal constituents. The environmental samples varied in collection size and analysis procedures as well. For example, 119 food samples were collected from endemic areas and 32 from non-endemic areas, while 88 soil samples were collected from endemic areas and 41 samples were from non-endemic areas. There appears to be marked variation in the number of samples analyzed for biological and environmental media, and it is unknown whether the samples were linked for multimedia comparison, or whether an effort was made to include a smaller subset of sample groupings with full multimedia sample collection.

The authors do not discuss the justification for the selection of a varied and smaller multimedia sample size for the endemic and non-endemic cases. Since both the sample media and constituent types vary among samples, it is presumed that there are fewer linked multimedia environmental and biological samples that can be evaluated during data analysis. It is unknown how many samples are linked and contain all media (both biological and environmental), if any. Providing this additional sample identification information will allow for metals concentrations in biological samples to be compared to those in environmental samples. An approach that includes multimedia sampling and analysis consistency with geo-referencing allows for more rigorous comparative data analysis, multivariate linear regression modeling, or environmental risk analysis, and will also decrease the overall uncertainty associated with the metal concentration results.

### Chemical constituent selection

Jayatilake et al. state that multiple agents may play a role in the pathogenesis of CKDu. Heavy metals, most notably arsenic and cadmium, have previously been potentially linked with CKDu [33]. Jayatilake et al. tested the majority of biological and environmental samples for three heavy metals (arsenic, cadmium, and lead), along with a smaller sample set for additional heavy metals. In addition to heavy metals, pesticides are among the environmental chemicals that have been associated with renal damage. To address this issue, Jayatilake et al. determined a suite of 11 mostly organochlorine pesticide residues in urine samples. The use of these chemicals has been banned in Sri Lanka, but organochlorines are persistent organic pollutants (POPs) [34]. To build upon the efforts of Jayatilake et al., it will be important to expand the analyte list beyond POPs to include commonly employed, non-persistent pesticides, including organophosphates. The analysis of urine for organophosphate metabolites can be used to assess current exposure to these agrochemicals. In addition, future studies could benefit from incorporating a broader suite of trace element analytes in the various study matrices as well as water and soil hydrogeochemical parameters. These additional data will build on the work of Jayatilake et al. and place measured heavy metal data in context. Ideally, most of the analytes in the suite will be determined in all study matrices. In addition, it is important to make available information about the percentage of the determined metals in soil available for uptake in both endemic and non-endemic areas.

In some instances, it may also be desirable to establish the chemical form or species of a heavy metal contaminant. For example, inorganic forms of arsenic, typically found in drinking water, are known to be more toxic than the organic forms of this element, often found in fish. Arsenic speciation in a subset of the urine samples will help to identify the source of arsenic exposure. The concentration, availability, and biological distribution of metals can also be significantly impacted by other hydrogeochemical considerations. Both soil geochemistry (pH, organic matter, the presence or absence of iron, zinc, and other metals, particle size speciation) and water hydrogeochemistry (pH, the presence or absence of fluoride, calcium, and other metals) data will be useful in determining the extent of heavy metal availability and uptake during future studies [35-37].

### Field documentation

Although the authors state which media were sampled for the study, detailed field documentation in Jayatilake et al. is not provided. As part of any subsequent publication based on the Sri Lanka MoH and WHO study, additional field method documentation detailing the sample media types listed below will help to develop conceptual site models, identify potential environmental hazards in the surrounding area, and interpret the overall analytical results. The types of field documentation that would be useful are discussed by media type below.

• *Soil* – Agricultural soils can be potential sinks for heavy metals, in large part due to agrochemical use [38], but heavy metal mobility and persistence within the soil column are dependent on many factors. Supplemental information regarding the employed soil sampling procedure (sample depth, collection pattern, use of composites, drying, sieving, and grounding) and soil physiochemical characteristics (soil type, location on property, proximity to roads) in endemic and non-endemic areas will be useful to fully interpret the study results. Moreover, the hydrogeochemistry associated with seasonally changing rice paddies can cause the mobilization or precipitation of heavy metals, depending on oxidation-reduction potential, thus sample timing in reference to paddy cultivation is of interest [39,40].

• *Groundwater* – The authors report that CKDu occurs in areas where groundwater is the primary source of drinking water, and that groundwater in the endemic areas is known to contain elevated levels of calcium and fluoride. Additional information about the collection of analyzed water samples, including calcium and fluoride levels, pH, the presence of organic matter, and other physiochemical properties, will help place heavy metal data in context. The concentrations of many heavy metals in water are known to vary temporally and spatially, so the timing and depth of water sample collection is an important point of interest. In addition, other investigators will benefit from dissemination of procedures used for sample handling, such as filtration or acidification to limit microbial growth prior to the digestion and analysis.

• *Food* – Jayatilake et al. collected an assortment of food (rice, pulses, vegetables, and fish), tobacco, pasture, and weed samples for heavy metal analysis. Information regarding how samples were collected and processed prior to the acid digestion (described in supplemental materials) is of interest. Plant species, including cultivars, are known to differentially bioaccumulate metals in roots, leaves, and shoots, thus information about the plant structures and cultivars analyzed will help to place the heavy metal data in context [41]. For example, unpolished brown rice has been reported to contain higher heavy metal levels than polished white rice. Similarly, fish are known to bioaccumulate metals in different tissues, so an understanding of what fish tissues were tested and how fish were harvested will facilitate interpretation of the results. The presence or absence of other trace elements in food samples and the general nutritional status of cases and controls (malnutrition, BMI, etc.) will minimize uncertainties and allow for a more expansive interpretation of the results.

• *Fertilizers* – The authors’ decision to analyze heavy metal concentrations in phosphate fertilizer is sound, as numerous literature reports link heavy metal contamination to the application of these products. However, cadmium or other metal concentrations levels will depend on the type of phosphate fertilizer. For example, rock phosphate, diammonium phosphate, and triple superphosphate may exhibit significantly different heavy metal impurity profiles [42,43]. Consequently, data regarding the type, source (e.g. a local mine or internationally), and application of the phosphate fertilizer tested is important to review. Organic soil amendments and pesticides may also contain metal impurities, information about their prevalence and use in endemic and non-endemic areas will be highly relevant.

• *Biological Samples –* From the article and supplemental material, it is not clear how urine or serum creatinine values were determined. In the case of urine analysis, this is of interest because data are creatinine-corrected. For the determination of metals in hair and nail matrices, removal of exogenous contamination is critical. The cleaning procedure described in the supplemental materials appears adequate to address this issue, but additional information about sample collection (distance of hair from scalp, single or multiple fingernail or toenail samples, etc.) will add greater understanding. For the analysis of serum, information about blood and serum collection tubes is important as their anticoagulants can contain trace metals as a function of the tube manufacturer batch lot number.

As described by media type above, the published paper and supplemental materials do not specifically document how the biological and environmental samples were collected, handled, stored, and transported, or specify that appropriate standard operating procedures (SOPs) and quality assurance/quality control (QAQC) guidelines were followed during fieldwork and transport for laboratory analysis. Although the article appendix provides information on sample preparation, collection procedures and protocols are omitted, making it difficult for others to use this information or build on the current study. Additional information on field procedures that address the following questions will enhance understanding of the study design and benefit other investigators in the field: Where were the environmental samples obtained from? Who collected the samples? What lateral and vertical location were soil samples collected from? Do soil samples constitute a grab sample or a composite sample from the area surrounding an individuals’ residence? What containers were the samples stored in? How was sample collection recorded? Was a chain-of-custody document used throughout the process to validate the integrity of the samples?

### Laboratory analytical methodologies

Jayatilake et al. employed sensitive analytical techniques for the determination of study analytes. Inductively coupled plasma mass spectrometry (ICP-MS) and graphite furnace atomic absorption spectrometry (GFAAS) were used for the determination of trace elements, while liquid chromatography with tandem mass spectrometry (LC-MC/MS) and gas chromatography with mass spectrometric detection and tandem mass spectrometric detection (GC-MS and GC-MS/MS) were used for determination of pesticide residues. For the ICP-MS measurements, a laboratory quality control regiment is described that included use of internal standards, interspersed analysis blanks, intra and inter-run precision through dilution verification and duplicate preparation, and importantly inclusion of standard reference materials (SRMs). However, it is not clear from the supplemental material if continuing calibration check samples were utilized or if preparation blanks were processed with the study samples (and if so, what analyte levels in these blanks were in comparison to the study samples). Although SRM data were reported, it was not possible to determine how many replicates were processed, the corresponding measurement precision, or how many replicates were used for establishing intra and inter-assay precision. Overall, it does not appear that a high-percentage of quality control (QC) samples was processed with study samples.

ICP-MS limits of detection (LODs) were provided for arsenic, cadmium, and lead in water and urine and also in digestion fluids, but information on how these limits were established was not provided. Some of the reported data appeared to have determined concentrations that fell below reported LODs, so it is not clear if multiple analytical batch-specific LODs were employed. The supplemental material also did not describe how values with determined concentrations below their respective LOD were treated statistically. The QC procedures for the GFAAS measurements used to determine selenium and other elements in serum samples were also not described. Additionally, the authors state that validated LC-MS/MS, GC-MS, and GC-MS/MS methods were utilized to determine the concentration of pesticide residues in urine samples, but information about how these methods were validated was not provided. The term ‘validated method’ has a very specific meaning for bioanalytical measurements, and intra and inter-assay precision and accuracy, linearity, matrix impact, specificity, and stability under scenarios is required. The scientific community will benefit from the provision of additional QC sample data to verify method performance.

Additional insights will be gained from determining the concentrations of a suite of other elements that can impact cadmium solubility and toxicity (for example, selenium, calcium, iron, zinc, and fluoride) in all applicable study matrices. The determination of inorganic and organic arsenic species in urine will help to identify the primary source of arsenic exposure. Residues from a suite of predominantly organochlorine pesticides were determined in urine samples and heavy metals were measured in some phosphate fertilizers, but additional information about agricultural practices, including specific agrochemical use, and about CKDu case and control nutritional status, diet, and drinking water sources will be useful in interpreting results.

### Conclusions and recommendations

Overall, the Sri Lanka MoH and WHO study represents one of the first multifaceted efforts to identify the risk factors of CKDu in Sri Lanka. With the publication, Jayatilake et al. brought greater attention and understanding to the emerging CKDu crisis in Sri Lanka. Their investigation concluded that multiple agents, including chronic exposure to low levels of cadmium, and genetic predisposition may play a role in the genesis of CKDu. The article presents data and findings that make an effort to address the potential environmental influences on CKDu prevalence. However, there are still many questions that remain unanswered.

Based on the critical analysis of the Sri Lankan MoH WHO study, we recommend the following:

1) *Further evaluate the CKDu case definition and refine medical testing procedures, as needed* – For the benefit of future studies, it is important that Jayatilake et al. detail the sensitivity and specificity of their CKDu medical testing procedures and indicators. Additionally, testing procedures could be further refined during future studies. NGAL may be a more sensitive marker for early evidence of kidney damage in CKDu compared to ACR. Additionally, it is recommended to exclude patients based on their previous history of infections known to be associated with GN (e.g. sore throat), rather than to exclude based on history of GN per se.

2) *Geospatially link biological and environmental samples* – The study deployed geospatial tools in the research and data collection, but Jayatilake et al. do not use GIS in their paper to geospatially link biological and environmental samples, or interpret their results. Publication of this GIS work will be valuable for future research. Furthermore, mapping heavy metal concentrations in biological and environmental samples both temporally and geospatially will support the identification of potential CKDu risk factors. The biological uptake and distribution of cadmium and other environmental nephrotoxic compounds can ultimately be influenced by several confounding factors, including soil and water physicochemical characteristics. This mapping is especially critical in understanding potential differential exposures in endemic and non-endemic areas with variable lithology.

3) *Provide more comprehensive information on the overall study design and field protocols* – Full dissemination of study methods, including population sampling design, multimedia sample selection and collection procedures, chemical constituent selection, and field documentation will permit the repetition of a subsequent study in Sri Lanka or elsewhere. Major data gaps in the published record associated with the study include, but are not limited to, the absence of an initial screening stage to select geographic sampling locations and study participants; incomplete information on how the biological and environmental samples are linked; a lack of physicochemical characterization for the environmental samples; no information on georeferencing of field samples and how georeferencing affected the strength of the results; and, a lack of information on the eating habits and environmental surroundings of the study participants. For example, soil depth, sampling collection and sieving type, and rice type all are important in understanding the distribution and uptake of heavy metals.

4) *Provide more comprehensive information on the laboratory protocols* – Although Jayatilake et al. employed sensitive analytical techniques throughout their investigation, additional detail concerning procedures for the establishment of detection limits, for the statistical treatment of analyte values flagged as “< LOD”, and for intra and inter-laboratory QC practices will be necessary to replicate the study. The authors do not explicitly mention this information or discuss the implications on study results. Publication of the study protocol, including all SOPs for sample selection, collection, handling, transport, and analysis will help future work on CKDu.

5) *Publish additional WHO studies on CKDu in Sri Lanka and integrate findings* – Additional publications of studies on CKDu in Sri Lanka funded by the WHO are encouraged. Informational bulletins previously issued by WHO outlined that this study was one of several sub-studies that were completed and funded on behalf of the WHO. This study, which was identified by WHO as the “Population Prevalence Study” covered similar concerns but used a different design approach than the “Hospital Registry Study” discussed by WHO [12,44]. It is important to know whether the results of this second study shed further light on the risk factors associated with CKDu and corroborate findings in the population prevalence study. It will be extremely valuable for the results of both studies to be integrated and disseminated in another peer-reviewed article.

In conclusion, Jayatilake and the entire research team are to be commended for drawing attention and greater understanding to this complex and critical public health crisis. With additional information and publications, the scientific community will be in a stronger position to build on the foundation laid by Jayatilake et al. and other investigators who continue to work to identify the aetiology of CKDu in Sri Lanka. Further, documentation of study protocols and findings will be useful in mitigating risks in Sri Lanka and in similar settings globally where CKD of unknown aetiology is increasingly becoming a public health issue.

## Response

By Shanthi Mendis

Email: mendiss@who.int

Address:

Senior Adviser Noncommunicable Diseases,

World Health Organization, Geneva, Switzerland

Although the problem of chronic kidney disease of uncertain aetiology (CKDu) had been recognized since the late 1990’s, a clear case definition and disease grades for CKDu was used for the first time in the Ministry of Health/World Health Organization National Research Initiative, published in 2013 [13]. We defined a CKDu case as an individual identified with an albumin-to-creatinine (ACR) ≥ 30 mg/g urine during initial visit and at a follow-up visit, a normal glycosylated hemoglobin (HbA1c <6.5%), not on treatment for diabetes, non-elevated blood pressure, and no past history of kidney disease or snake bite. Redmon et al. [45] now suggest that more sensitive markers for kidney damage should be used in the case definition. For research purposes, it is not at all difficult to revise the case definition using more sensitive markers of kidney damage, including estimated glomerular filtration rate and urinary neutrophil gelatinase-associated lipocalin-to-creatinine ratio. Indeed, when the case definition was developed, more sensitive parameters were given due consideration but they were not included in the definition for several important reasons. Firstly, there were many logistic difficulties in applying more sensitive parameters in a population based study conducted in a resource constrained setting. Secondly, a case definition with more sensitive parameters was not cost effective. There was another important consideration. The case definition that was developed also needed to be suitable for population screening, service planning and delivery purposes after the research was completed. Given the resource limitations, applying even the present case definition for screening purposes will continue to be a challenge.

Redmon et al. [45], also suggest that the blood pressure cut off in the definition should be lowered to 140/90 mm Hg. The population prevalence of mild hypertension is high in Sri Lanka [46]. If the blood pressure cut off is lowered, there is a likelihood that many potential CKDu patients would be eliminated from a study.

We also do not agree with Redmon et al. [45], that patients should be excluded based only on a previous history of infections known to be associated with glomerulonephritis rather than on a documented history of glomerulonephritis. Given the limitations in health information systems in Sri Lanka, such a history will be difficult to ascertain. Further, when the histological features of CKDu is known to be different from glomerulonephritis, there is no reason to include documented cases of glomerulonephritis as potential CKDu cases [1].

Due to the scarcity of published evidence, at the time our study was conducted, a wide range of biological (e.g. urine, blood serum, hair and nails.) and environmental samples (water, rice, different types of vegetables and fish, tobacco, fodder, pesticides, fertilizer and soil) had to be analysed in an effort to link the biological basis of CKDu with potential environmental risk factors. At the same time we also needed to use sensitive analytical techniques to ensure accuracy and reliability of data. These analyses were costly and with the available resources it was not possible to increase the sample sizes and link multimedia samples. For example, it was not feasible to analyse biological samples of all CKDu cases for all heavy metals.

We have provided salient information on how the biological and environmental samples were collected, stored, and transported and on laboratory analytical methodologies in the paper and supplementary files and appendices. More information including protocols could have been provided if we published the large body of data in several different papers. However it was felt that a comprehensive paper with all relevant data will better serve the purpose of informing policy, which was the primary objective of the Ministry of Health/World Health Organization study.

Finally, the critical review of Redmon et al. [45] does not invalidate any of our findings which can form the basis for policy change. We agree with Redmon et al. [45], that further research needs to be done, particularly research in actionable areas which can provide affordable and pragmatic solutions for addressing CKDu [47]. However, planning and executing such research should not in anyway delay immediate policy level actions that are being taken by the interministerial committee appointed by the President of Sri Lanka to address this important public health issue [48].

## Pre-publication history

The pre-publication history for this paper can be accessed here:

http://www.biomedcentral.com/1471-2369/15/125/prepub
